# The application progress of artificial intelligence in the diagnosis of Alzheimer’s disease

**DOI:** 10.3389/fneur.2026.1852128

**Published:** 2026-10-01

**Authors:** Enqi Liu, Meijia Cheng, Yetao Ju, Yi Ru, Yongming Liu, Dongyu Min

**Affiliations:** 1 Liaoning University of Traditional Chinese Medicine, Shenyang, China; 2 Liaoning University of Traditional Chinese Medicine Affiliated Hospital, Shenyang, China

**Keywords:** Alzheimer’s disease, artificial intelligence, early diagnosis, imaging data analysis, multi-omics analysis

## Abstract

Alzheimer’s disease (AD) is a progressive neurodegenerative disorder, where traditional diagnostic methods are constrained by limited sensitivity and restricted clinical utility. This review synthesizes recent applications of artificial intelligence (AI) in AD diagnostics, focusing on machine learning (ML) and deep learning (DL) across neuroimaging modalities and multi-omics research. AI algorithms can automatically extract subtle microstructural, metabolic, and molecular features, enabling high-precision classification of AD stages, patient stratification, and non-invasive early screening through multimodal data integration. However, translating AI into routine clinical practice still faces critical challenges, including high equipment costs, insufficient model generalizability across real-world cohorts, data class imbalance, and complex cross-platform standardization barriers. Future research should prioritize cost-effective surrogate modeling, robust multi-center validation, dynamic longitudinal integration, and mechanistic interpretability to accelerate the standardized clinical translation of AI-driven AD diagnostics.

## 1 Introduction

AD is the most common form of age-related dementia and is a progressive neurodegenerative disorder. Its main manifestations include cognitive decline, abnormal behavior and mood changes, and loss of daily living abilities (1). The pathological features of AD include the deposition of β-amyloid (Aβ) plaques, abnormal accumulation of phosphorylated tau protein, and neuronal degeneration (2). Currently, approximately 50 million people worldwide are affected by this disease (3), and it indirectly impacts tens of millions of families, who endure significant emotional and daily life burdens as their loved ones gradually lose cognitive abilities over the years. Despite continuous advances in scientific research, the diagnosis and treatment of AD still face many challenges. Traditional diagnostic methods include neuropsychological assessments, imaging examinations, and biomarker testing (4). Neuropsychological scales such as MoCA and MMSE help assess cognitive function but can only detect impairments when the condition is advanced (5). Imaging techniques, including magnetic resonance imaging (MRI), positron emission tomography (PET) scans, and others, can detect structural and functional changes in the brain, but they have limited sensitivity in the early diagnosis of AD (6). Concurrently, the emergence of highly sensitive, blood-based biomarkers—such as plasma p-tau217—has recently opened new avenues for minimally invasive clinical screening (7). However, fully unlocking the predictive synergy between these micro-level blood biomarkers and macro-level neuroimaging data remains a significant challenge for human clinicians. To bridge this multimodal gap, AD diagnosis is undergoing a transformative shift driven by the rapid advancement of AI. As illustrated in Figure 1, this integrated framework showcases how AI serves as a methodology core, bridging the chasm between micro-level biological data and macro-level neuroimaging to enable early diagnosis.

**Figure 1:**
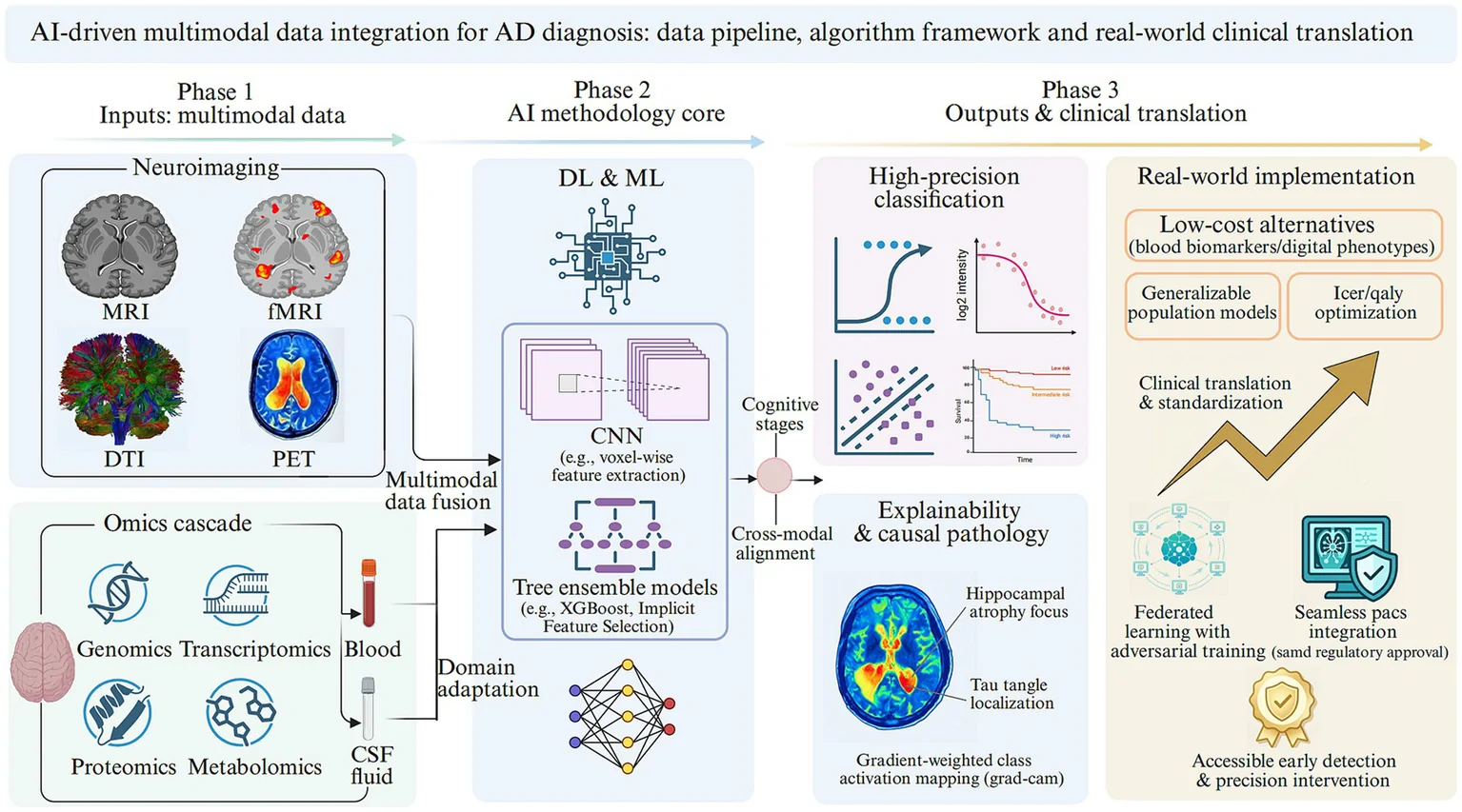
AI-driven multimodal data integration for AD diagnosis: data pipeline, algorithm framework, and real-world clinical translation. The framework illustrates the progression from Phase 1 data acquisition (coupling neuroimaging with multi-omics cascades through data fusion and domain adaptation) to Phase 2 methodological core processing (employing CNNs and tree ensemble models for cross-modal alignment). Phase 3 demonstrates the downstream clinical translation, bridging high-precision, explainable diagnostic outputs (such as Grad-CAM pathology localization) with real-world deployment strategies, including federated learning, ICER/QALY optimization, and seamless PACS integration for precision intervention.

AI refers to a type of technology that enables computer systems to make autonomous decisions and solve problems by simulating human thinking, learning, and reasoning processes (8). AI technology encompasses multiple fields, including ML, DL, natural language processing, computer vision, and speech recognition (9). These technologies are continuously developing and advancing, driving innovation and transformation across various industries. Among them, ML, an important branch of AI, focuses on developing computer programs that can improve themselves through experience. By analyzing vast amounts of data, ML algorithms can identify underlying patterns and make predictions or decisions (10). DL is a more specialized area within AI and ML. DL models can mimic the structure and function of human brain neurons, and through multi-layered neural networks, they can automatically extract complex features from vast amounts of medical imaging data, enabling disease diagnosis and prediction without human intervention (11). In the medical field, especially in the early diagnosis and prediction of neurodegenerative diseases such as AD, DL and ML have demonstrated remarkable capabilities (12). Traditional medical imaging techniques, such as MRI and PET, can provide doctors with direct information about the structure and function of the brain, but they still have limitations in detecting subtle changes or early lesions. AI, through deep analysis of neuroimaging data, can overcome these limitations and offer a more precise assessment of the brain’s structure and function (13). It can reveal structural changes in the brain, such as atrophy in specific regions or functional decline, and help predict the onset and progression of diseases like Alzheimer’s. In summary, AI is driving AD diagnosis toward greater intelligence, personalization, and precision, and in the future, it will play a key role in improving diagnostic accuracy, optimizing treatment, and enhancing patients’ quality of life, benefiting more individuals.

To ensure methodological transparency and reproducibility, a structured literature search was conducted across PubMed and Web of Science databases up to June 2026, using combined search terms for AD (“Alzheimer’s disease,” “mild cognitive impairment”), AI algorithms (“deep learning,” “machine learning”), neuroimaging (“magnetic resonance imaging,” “positron emission tomography”), and multi-omics (“proteomics,” “genomics,” “metabolomics”). Included studies comprised peer-reviewed original research and reviews utilizing AI for AD diagnosis that reported explicit methodologies, quantitative evaluation metrics (e.g., area under the curve (AUC), accuracy), and validation strategies. Exclusion criteria applied to non-English publications, conference abstracts, and studies lacking robust technical validation or AD-specific research cohorts. Two reviewers (NL and MC) independently conducted title/abstract screening and full-text eligibility evaluations, with any discrepancies resolved through consensus with a senior corresponding author (DM), prioritizing high-impact pioneering studies published within the past 5 years. By critically analyzing algorithmic applications within the neuroimaging and multi-omics domains, this review aims to provide researchers and clinicians with a systematic and objective perspective, clearly delineating the current research landscape, technical bottlenecks, and future trajectories of AI in AD diagnostics.

## 2 Imaging data analysis

AI has become a core tool for neuroimaging analysis, thoroughly revolutionizing the clinical diagnostic paradigm for AD. Advanced ML and DL frameworks can efficiently process high-resolution neuroimaging data, and capture focal, subtle, and occult pathological changes that are difficult to identify via manual clinical image reading. By conducting voxel-level and region-of-interest-based precise analysis of brain injury-susceptible regions including the hippocampus, corpus callosum, and thalamus, AI models can detect early biomarkers of neurodegenerative lesions in advance, achieving precise early screening and diagnosis of AD (14, 15).

### 2.1 The application of AI in MRI

MRI serves as a cornerstone of the clinical diagnosis of AD, and it can clearly depict changes in the macroscopic structure of the brain. Among these changes, hippocampal atrophy, which is closely linked to memory and cognitive function, is one of the most characteristic neuroimaging findings in early AD (16). In addition to the hippocampus, progressive degenerative changes in the frontal and temporal lobes also serve as key evidence for MRI-based detection of AD pathology (17). In recent years, the deep integration of AI with multimodal MRI technology, which integrates multi-sequence data including structural MRI (sMRI), functional MRI (fMRI), and diffusion tensor imaging (DTI), has significantly improved the accuracy of differential diagnosis between AD and mild cognitive impairment (MCI) (18).

sMRI remains the clinical gold standard for the quantitative assessment of regional neurodegeneration, particularly hippocampal and cortical atrophy (19). Möller et al. (20) employed a support vector machine (SVM) model based on T1-weighted gray-matter-density maps to discriminate AD from behavioral variant frontotemporal dementia (bvFTD). Using an independent test set, the model achieved an 88% diagnostic accuracy, thereby confirming the effectiveness of traditional machine-learning approaches for structural-imaging analysis. However, classification models that rely solely on volumetric features tend to show relatively poor diagnostic performance when faced with atypical clinical cases and subtle early structural variations. To evaluate the real-world generalization performance of AI diagnostic models, Kang et al. (21) conducted a large-scale external validation of the 3D Nested hierarchical Transformer (3DNesT) model using a multicenter cohort of 3,258 scans, achieving a cross-validation baseline accuracy of 90.0%. Notably, subgroup analyses revealed key neuropathological findings: a subset of AD patients exhibited severe Aβ and tau protein deposition without obvious macroscopic brain atrophy. This finding indicates that macroscopic brain structural degeneration significantly lags behind molecular-level pathological damage, making it difficult for models relying solely on traditional macroscopic volumetric features to identify occult AD cases with no apparent atrophy. To overcome this limitation, recent research has shifted its focus to Vision Transformers (ViTs) and self-supervised foundation models [e.g., 3D Masked Autoencoders (3D-MAE)]. Such models can break through the inherent local inductive bias of conventional convolutional neural networks (CNNs), capturing global anatomical associations and high-frequency fine microstructural features from massive unlabeled MRI cohorts (22). For example, Ergün et al. (23) evaluated the self-supervised pretraining performance of 3D-MAE and Joint Embedding Predictive Architectures (JEPA) on multicenter unlabeled MRI data; by incorporating frequency-domain reconstruction optimization into the MAE framework, the model can accurately capture subtle structural variations in the early brain, thereby effectively identifying preclinical AD cases that have no obvious brain atrophy but already present molecular pathological deposition. This result not only validates the application value of frequency-domain enhanced self-supervised representations in preclinical AD screening, but also provides a novel algorithmic paradigm for overcoming the pathological lag effect of sMRI. Beyond single-modal optimization, overcoming the inherent pathological lag of sMRI requires capturing functional and microstructural anomalies before macroscopic atrophy emerges. By integrating high-dimensional radiomic textures with dynamic functional connectivity, advanced AI algorithms can accurately detect occult molecular signals mapped onto macroscopic imaging. Demonstrating this structural-functional synergy, Itkyal et al. (24) developed a deeply fused multimodal CNN model that integrates gray matter structural features from sMRI and whole-brain functional atlases derived from resting-state fMRI (rs-fMRI), achieving a classification accuracy of 94.12% on the Alzheimer’s Disease Neuroimaging Initiative (ADNI) dataset. This result validates the prominent advantages of structural-functional multimodal fusion in the early diagnosis of AD. However, traditional CNNs are limited by their local receptive fields, unable to efficiently model cross-modal, long-term temporal global dependencies, and thus have an obvious performance ceiling. In recent years, research efforts have extended ViTs and self-supervised foundation models to the field of multimodal fusion, leveraging their powerful long-range association modeling capabilities and the benefits of unsupervised pre-training to further enhance the representational depth for fine-grained multimodal structural and functional abnormalities (25).

fMRI, which relies on blood oxygen level-dependent (BOLD) signals, can reflect cerebral neural activity and metabolic dysfunction in real time, making it a core technology for capturing early functional abnormalities that precede the onset of structural brain lesions (26). Traditional manually constructed functional connectivity matrices suffer from high-dimensional redundancy, low stability, and high computational costs. To address these limitations, Farhan et al. (27) developed an end-to-end spatiotemporal DL framework that integrates CNNs and long short-term memory networks (LSTMs). This framework eliminates the need for manual feature selection, and can automatically encode spatial features and capture temporal dynamic information from neuroimaging data, achieving 99% accuracy in multi-stage AD progression staging tasks. However, from a clinical translation perspective, such near-perfect accuracy carries significant methodological limitations. The signal-to-noise ratio of BOLD-based connectivity matrices is inherently low, rendering them highly vulnerable to interference from physiological artifacts including subtle patient head motion, respiration, and cardiac signals. The strong performance of existing models depends heavily on standard benchmark datasets such as the ADNI, which have undergone expert-level rigorous denoising, artifact regression, and motion correction. In real-world clinical settings, by contrast, patient compliance varies widely, resulting in a marked increase in artifacts, scanner heterogeneity, and spatiotemporal noise. Additionally, implicit data leakage during preprocessing—such as conducting feature selection or normalization on the entire dataset before splitting for cross-validation—often leads to overly optimistic performance reporting. These models frequently suffer performance collapse when deployed prospectively in clinical practice for out-of-distribution (OOD) cases. Beyond the challenges posed by cross-sectional imaging artifacts, the irregularity of longitudinal follow-up data further limits the clinical translation and deployment of such models. To accommodate the complexity of real-world clinical data, Moghaddami et al. (28) abandoned conventional regular convolution operations and adopted a modeling paradigm based on non-Euclidean topology, developing a temporal model that combines graph neural networks (GNNs) with recurrent neural networks (RNNs). The model was specifically designed for real-world clinical scenarios featuring irregular follow-up intervals and missing data, and it achieved an accuracy of 68.8% on the challenging task of predicting conversion from cognitively normal (CN) to MCI, thereby validating the clinical utility of temporal topology models in dynamic brain network analysis. Although time-series topological models can accommodate the properties of longitudinal data, AD progression exhibits extremely high individual heterogeneity, which renders traditional coarse-grained staging models unable to meet the demands of precision clinical diagnosis and treatment. However, single-modality functional neuroimaging alone cannot fully capture the extreme individual heterogeneity of AD progression. To bridge single-modality functional analysis with comprehensive patient profiling, Ren et al. (29) developed an interpretable multimodal framework that integrates 3D MRI images with clinical assessment metrics, enabling progression subtype classification for AD patients and individualized cognitive trajectory prediction at 6, 12, and 48 months. By incorporating an attention mechanism into the multimodal LSTM network, the model achieved MAE as low as 0.196, 0.203, and 0.261 on prediction tasks at 6, 12, and 48 months, respectively, with corresponding prediction accuracies of 0.903, 0.845, and 0.791. SHAP interpretability analysis confirmed that memory- and language-related imaging and clinical features are core drivers of disease progression prediction, which aligns highly with the underlying pathological mechanisms of AD-associated cognitive impairment (30). Additionally, to overcome the limitations of traditional binary and ternary classification models and address the challenge of refined clinical staging diagnosis, Jahan et al. (31) developed an interpretable multi-class prediction framework that integrates clinical scales, MRI segmentation features, and neuropsychological assessment data. This framework enables fine-grained differentiation across five categories: CN, AD, non-AD dementia, uncertain dementia, and other dementia. Results from 10-fold cross-validation on the OASIS-3 dataset showed that the random forest model demonstrated the best performance, with an accuracy of up to 98.81%. These findings confirm that multimodal data fusion can significantly expand the applicability of AI models in refined clinical staging and stratified patient management.

By quantifying the anisotropy of water molecule diffusion, DTI can assess the microstructural integrity of cerebral white matter tracts, enabling the early detection of microstructural pathological changes such as myelin damage and axonal disruption before macroscopic atrophy becomes detectable on sMRI (32, 33). Marzban et al. (34) developed a lightweight shallow CNN that integrates DTI and structural imaging metrics for AD/MCI staging diagnosis. The model achieved a classification accuracy of 93.5% (AUC = 0.94) in distinguishing AD patients from healthy controls, and demonstrated excellent stability in repeated-scan validation, making it well-suited for longitudinal clinical research scenarios. To further leverage the complementary advantages of multimodal data, address gradient decay in unimodal models, and enhance their interpretability, Li et al. (35) developed a lightweight dual-branch ResNet-10 model named MADNet. The model achieves dynamic parallel fusion of macroscopic volumetric features derived from sMRI and microscopic diffusion injury features from DTI, and incorporates a cross-modal attention (Cross-Modal Attention) mechanism to optimize decision weights. In validation experiments, the model achieved a classification accuracy of 96.54% (AUC = 0.9597) for distinguishing AD patients from healthy controls, and an early warning accuracy of 83.33% for MCI (specificity = 0.9347). Visual validation based on Grad-CAM showed that the model’s decision regions were highly consistent with classic pathological regions such as cortical atrophy and lateral ventricular enlargement, providing a reliable pathological basis for the model’s clinical deployment. Although diagnostic paradigms that fuse sMRI-derived macroscopic features and DTI-derived microscopic white matter injury indicators demonstrate significant advantages, their clinical translation still faces major bottlenecks: on the one hand, multimodal fusion requires ultra-high-dimensional feature alignment, cross-modal attention mechanisms, and nonlinear tensor registration to eliminate non-biological batch effects across scanners (e.g., data normalization via the ComBat method); on the other hand, the management of missing modalities in patients with incomplete imaging sequences remains a pressing practical challenge that needs to be addressed via generative imputation or dynamic masking architectures.

### 2.2 The application of AI in PET

PET imaging visualizes cerebral metabolic activity and molecular distribution via radioactive tracers, and serves as the core imaging modality for AD pathological detection (36). In the 2024 updated diagnostic criteria for AD jointly released by the U.S. National Institute on Aging (NIA) and the Alzheimer’s Association (AA), Aβ and tau protein deposition are explicitly designated as core diagnostic biomarkers for AD (37). In this regard, PET can accurately capture the spatial distribution and burden levels of the accumulation of these two core pathological proteins at the early stage of disease, thereby enabling early detection of pathological alterations. Choi et al. (38) developed an automated deep CNN (DCNN) analysis system based on 3D PET imaging to predict the progression from MCI to AD. On the ADNI dataset, the system achieved a predictive accuracy of 84.2%, significantly outperforming conventional analytical methods. Additionally, the model’s predictive results were highly correlated with changes in patients’ cognitive function, demonstrating the immense potential of DL in uncovering neuroimaging biomarkers for AD. However, this study heavily relies on the standardized ADNI dataset, and the model’s generalizability on heterogeneous real-world clinical imaging data still requires further validation.

In addition to the limitations in model generalizability, the high cost of PET equipment, expensive examination fees, and the high threshold of auxiliary analytical software severely hinder the widespread clinical application of high-accuracy AI-PET diagnostic technologies (39). To overcome the aforementioned cost barriers, researchers have initiated investigations into substituting routine clinical indicators for high-end PET examinations. Jasodanand et al. (40) integrated data from 7 cohorts comprising a total of 12,185 participants to develop a multi-omics computational framework that uses routine neurological assessment indicators to predict PET-derived molecular signatures. When classifying the status of Aβ and tau proteins, the framework achieved AUROC values of 0.79 and 0.84, respectively, and is applicable to rapid screening of clinical trial participants. However, the model heavily relies on the harmonized standardization of multi-center data, and controlling data heterogeneity remains challenging; therefore, its applicability in unscreened real-world community-dwelling populations is constrained. To further explore the feasibility of replacing tau-PET examinations with low-cost, non-invasive biomarkers, Karlsson et al. (41) integrated clinical variables, plasma biomarkers (including p-tau181 and p-tau217), and sMRI data to develop a predictive framework for continuous tau-PET tracer binding burden. When evaluated as a continuous scalar regression task, the model incorporating plasma biomarkers accurately predicted overall global tau-PET retention, achieving a coefficient of determination (*R*^2^) ranging from 0.66 to 0.69, whereas sMRI features provided optimal predictive performance for regional hemispheric asymmetric lesions (*R*^2^ = 0.28–0.42). Importantly, while these regression outputs (*R*^2^) effectively quantify continuous molecular pathology, translating such algorithms into clinical screening requires dichotomizing predicted values into actionable clinical cutoffs. From a clinical translation perspective, the positioning of such AI-driven surrogate models is not to completely replace gold-standard PET imaging, but rather to serve as highly sensitive “rule-out” screening tools in primary care settings. By accurately identifying individuals with a low probability of amyloid or tau pathology using accessible blood biomarkers and routine clinical data, these algorithms can act as a crucial triage step, significantly reducing unnecessary PET scans. However, realizing this potential in practice remains constrained by three major hurdles: the lack of cross-platform standardization for plasma assays, severe class imbalance in real-world screening cohorts (which risks high false-negative rates when thresholding), and the inability of static cross-sectional models to capture the longitudinal, trans-synaptic propagation dynamics of tau pathology.

## 3 The application of AI in AD Omics

Biomarkers for AD are primarily derived from peripheral blood, cerebrospinal fluid (CSF), and neuroimaging data (42). In recent years, the rapid advancement of high-throughput sequencing and mass spectrometry technologies has driven the systematic application of multi-omics technologies (including genomics, transcriptomics, proteomics, and metabolomics) in AD pathological research, which can precisely capture early molecular perturbations such as aberrant gene expression, metabolic dysregulation, and risk locus mutations associated with neuroinflammation and neurodegenerative cascades (43, 44). This approach also overcomes the sensitivity limitations of conventional enzyme-linked immunosorbent assay (ELISA) in detecting CSF Aβ and tau proteins, and provides a more comprehensive molecular basis for early AD pathological stratification (45, 46).

Single-molecule biomarkers face bottlenecks in diagnostic sensitivity and specificity, and cannot meet the needs of precision diagnosis for AD. Therefore, leveraging AI and ML to deeply mine high-throughput multi-omics data and develop multifeature combined diagnostic models has become a core direction for precision diagnosis of AD. In the study by Stamate et al. (47), 242 CN individuals and 115 AD patients were enrolled, and their plasma metabolomics data were used to systematically compare the performance of multiple ML algorithms. The results showed that the XGBoost model achieved the best diagnostic performance, with an AUC of 0.88, outperforming deep neural networks (AUC = 0.85) and random forests (AUC = 0.85). Its overall diagnostic efficacy was comparable to that of invasive CSF testing. This performance gap stems from fundamental structural differences between spatial imaging and tabular omics data. Unlike 3D medical images that align with local CNN priors, omics datasets consist of non-spatial scalar variables constrained by *N* ≪ *P* (small sample size, high feature dimensionality). Unconstrained deep neural networks lack translation invariance, making them prone to overfitting on tabular noise, whereas tree ensembles naturally excel through automatic feature selection and subspace sampling. However, this tree-versus-DL dichotomy is shifting. When incorporating domain-specific structural priors, GNNs mapped onto protein–protein interaction or metabolic networks overcome *N* ≪ *P* limitations by enforcing biological topology. Modern tabular architectures (e.g., TabNet) further enable DL to effectively capture high-order multi-omics interactions.

Given the strong performance of tree-based models on high-dimensional omics data, Yi et al. (48) developed an interpretable ML framework based on XGBoost-SHAP, aiming to address two key challenges in AD diagnosis: class imbalance and three-class classification (CN/MCI/AD). Instead of adopting resampling techniques that are prone to causing data leakage and overfitting, the study balanced the data distribution via algorithm-level sample weight adjustment. The model achieved excellent classification performance on both the ADNI and NACC datasets, outperforming all benchmark models (including random forest, Bagging, AdaBoost, naive Bayes, and unweighted XGBoost) across all evaluation metrics. Interpretability analysis based on SHAP identified CDRSB, ADAS13, and ventricular volume as risk factors for AD onset, and LDELTOTAL and MMSE as protective factors, with the impact patterns of these core features remaining consistent in the external validation cohort. This work effectively resolves the black-box limitation of traditional ML models and the class imbalance problem commonly encountered in clinical data.

Multimodal fusion and molecular subtyping analysis are driving omics-based intelligent diagnosis of AD beyond “single biomarker screening” toward “refined pathological stratification.” To address the limitation of insufficient representational capacity of unimodal genomic data, AlMansoori et al. (49) demonstrated the computational advantages of cross-modal complementary integration: by integrating blood-derived gene expression data, single nucleotide polymorphism (SNP) data, and clinical features, and combining mutual information-based feature selection with SVM, their multimodal fusion model achieved an AUC of 0.94, far outperforming unimodal gene expression models (AUC = 0.65) and SNP-only models (AUC = 0.63). Subsequent SHAP analysis further identified core genes associated with axonal myelination and synaptic vesicle membrane synthesis. These results demonstrate that heterogeneous data from different omics layers exhibit significant feature complementarity at the algorithmic level, which can effectively compensate for the drawback of weak signals in single-omics data.

Besides improving diagnostic accuracy, AI-driven multi-omics analysis also shows a unique advantage in revealing the high clinical heterogeneity of AD and driving precise subtyping. From the perspective of the immune microenvironment, Lai et al. (50) used weighted gene co-expression network analysis (WGCNA) combined with XGBoost (test set AUC 0.86) to identify five core immune feature genes (CXCR4, PPP3R1, HSP90AB1, CXCL10, S100A12). Through unsupervised clustering, the study successfully divided AD patients into two distinct subtypes—immune-active and metabolic phenotypes—and predicted targeted potential drugs for each (e.g., MK-866 and arachidonyl trifluoromethane), a result that was experimentally validated by RT-PCR. This work demonstrates that the application of AI in omics has moved beyond traditional binary classification, delving into pathological mechanism mining and individualized precision medication guidance. However, when translating high-dimensional omics models (particularly proteomics) into clinical practice, specificity bottlenecks and sampling invasiveness remain non-negligible practical obstacles. In the field of CSF proteomics, Bader et al. (51) identified via mass spectrometry that abnormal expression of proteins including tau, SOD1 and PARK7 is closely linked to glycolytic dysregulation; however, as glycolytic stress is a non-specific pathological response shared across multiple neurodegenerative diseases, single protein panels fail to deliver precise differential diagnosis. To overcome this specificity bottleneck, Hou et al. (52) leveraged data from 10 multinational cohorts, using ML to screen for a universal diagnostic panel comprising 12 CSF proteins, and achieved high-accuracy differentiation of AD, MCI, and frontotemporal dementia. While this model demonstrates strong cross-center generalization performance, its reliance on invasive lumbar puncture sampling still poses a major barrier to deploying omics AI models in large-scale community early screening. Future research urgently needs to shift its computational focus to non-invasive biofluid omics, such as peripheral blood, to strike a balance between high specificity and low invasiveness.

## 4 Summary and Outlook

AI has demonstrated significant advantages in the diagnosis of AD. Leveraging ML, DL, and large model architectures, AI can automatically extract high-dimensional features from multimodal imaging data including sMRI, fMRI, DTI, and PET, enabling early detection of occult lesions and prediction of disease progression. It also exhibits high sensitivity and accuracy in identifying MCI, differentiating dementia subtypes, and predicting trajectories of cognitive decline. Furthermore, integrating AI with multi-omics data (genomics, transcriptomics, proteomics, metabolomics) and peripheral fluid biomarkers has greatly advanced non-invasive, precise early-stage patient stratification.

Despite these strengths, the clinical translation of AI-assisted AD diagnosis still faces five major systemic bottlenecks. First, health economics and equipment access constraints limit widespread adoption: current high-precision AI models rely heavily on high-end PET scanners, advanced MRI sequences, and high-performance computing hardware, making them difficult to deploy in primary care institutions. In the absence of curative AD treatments, the cost-effectiveness of high-cost diagnostic approaches still requires further evaluation using metrics such as quality-adjusted life years and incremental cost-effectiveness ratios, and there is an urgent need to develop low-cost population screening systems that combine peripheral blood biomarkers with multimodal digital phenotypes. Second, poor generalizability and dataset bias undermine real-world performance: most existing models are trained on homogeneous single-center cohorts such as the ADNI and Open Access Series of Imaging Studies (OASIS), and suffer from obvious demographic bias, scanner variability, and unresolved OOD shift issues; when tested on real-world multi-center heterogeneous data covering diverse ethnicities, educational backgrounds, and cognitive reserve levels, model performance often drops sharply. Third, the black-box nature of models and limited interpretability hinder clinical trust: the coarse heatmaps generated by traditional attribution methods such as Grad-CAM are prone to noise interference and weight misallocation, so building trustworthy clinical decision support systems requires advancing to next-generation explainable AI (XAI) frameworks that integrate methods including Integrated Gradients, SHAP, LIME, cognitive and stochastic uncertainty estimation (to filter high-confidence false positives), and causal inference (such as structural causal models and counterfactual image reasoning) to ensure model predictions align with classic AD pathological mechanisms including the Aβ cascade hypothesis and tau protein propagation. Fourth, dilemmas in data privacy, foundation models, and regulatory pathways create deployment barriers: centralized data aggregation is strictly restricted by privacy regulations such as the General Data Protection Regulation (GDPR) and the Health Insurance Portability and Accountability Act (HIPAA); while distributed federated learning can protect data privacy, it still faces security risks from gradient reversal attacks that can reconstruct 3D brain structures. Recent advances in multimodal foundation models, self-supervised pretraining, and large language models offer new opportunities for zero-shot clinical reasoning and cross-center feature alignment, but when such technologies are deployed as software as a medical device (SaMD), they still need to comply with regulatory requirements from the FDA and EMA [such as Good ML Practice (GMLP) and Predefined Change Control Plans (PCCP)], and undergo strict validation for algorithmic bias, cybersecurity risks, and catastrophic forgetting during continuous learning. Fifth, insufficient clinical system integration and dynamic optimization lead to practical usability issues: existing AI models have poor compatibility with hospital Picture Archiving and Communication Systems (PACS) and Hospital Information Systems (HIS), and lack dynamic confidence filtering mechanisms that easily cause clinical alert fatigue; meanwhile, the current fixed regulatory model also restricts continuous iterative optimization of models based on real-world data.

To overcome these systemic bottlenecks, the field of AI-assisted AD diagnosis requires comprehensive paradigm upgrades at the levels of underlying algorithms, data architecture, clinical implementation, and regulatory frameworks. A health economics-oriented approach should be adopted, using lightweight technologies such as knowledge distillation and quantization compression to reduce hardware barriers and meet the needs of primary care settings. Unsupervised domain adaptation (UDA) and self-supervised contrastive learning methods should be introduced to eliminate non-biological batch effects and improve algorithmic fairness and generalizability in low-resource environments. The field should move beyond traditional post-hoc statistical attribution approaches, incorporating prior knowledge of brain anatomy and pathological cascades into GNNs and multimodal foundation models to achieve mechanism-driven interpretable modeling. A privacy security framework integrating federated learning, homomorphic encryption, and differential privacy should be established, with native compatibility with hospital systems via microservice APIs, and dynamic uncertainty estimation mechanisms should be introduced to suppress false positives and reduce clinical alert fatigue. The full-lifecycle regulatory system for SaMD should be improved to support continuous adaptive learning of models, ultimately achieving safe iterative development and closed-loop optimization of AI in ultra-early AD screening, precise patient stratification, and individualized prognosis.
