# Supplementary material for: The application progress of artificial intelligence in the diagnosis of Alzheimer’s disease
Source: Front Neurol. 2026 Oct 1;17:1852128. doi: 10.3389/fneur.2026.1852128 (PMC13669838; doi:10.3389/fneur.2026.1852128)
Supplement: Supplementary file 1 [file Table_1.docx]

Table S1. AI models and diagnostic performance for Alzheimer’s disease biomarkers.

| **Image modalities / Data types** | **Researchers** | **AI architecture / model** | **Dataset & Sample Size (*N*)** | **Diagnostic performance & key outputs** | **Limitations & Translational Bottlenecks** | **Evidence Grade (PROBAST)** | **Translational Confidence (TRL)** |
| --- | --- | --- | --- | --- | --- | --- | --- |
| sMRI | Möller et al. | SVM on T1-w GM density maps | (*N*＝229 total)  Training set: n＝115  Prediction set: n＝114 | (Independent Test) Accuracy:  •AD vs. HC: 88%  •bvFTD vs. HC: 85%  •AD vs. bvFTD: 82%  AUC Range: 0.81–0.95 | Volumetric classifiers perform poorly when faced with atypical clinical variants. | High Risk of Bias  (Single-cohort internal sample split; lacks prospective multi-center external validation) | TRL 3 (Low) |
|  | Kang et al. | 3DNesT and other CAD techniques | 5 multisite datasets & 2 multidisease datasets (*N*＝3,258 total baseline scans) | 10-fold Cross-validation •Accuracy: 90.0%±2.3%  •External Validation Accuracy: 82.2%, 90.1%, and 91.6% across three independent external datasets. | Structural atrophy lags behind downstream molecular pathology; blind to structurally occult AD phenotypes. | Low Risk of Bias  (Large *N* > 3,000, multi-center design with multiple independent external dataset validations) | TRL 5 (High) |
| sMRI + rs-fMRI | Itkyal et al. | Multi-channel CNN with multi-modality fusion | ADNI dataset  • 2-way tasks (*N*＝466): 83 AD, 383 CN  • 3-way tasks (*N*＝730): 83 AD, 264 MCI, 383 CN | AD vs. CN Classification: Achieved 94.12% test accuracy and an AUC score of 97.79. | High-dimensional registration and multi-branch deep alignment demand extensive computational power. | High Risk of Bias  (Validated solely on retrospective ADNI data without independent external clinical cohort validation; potential data leakage risk in fMRI network feature extraction) | TRL 3 (Low) |
| fMRI | Farhan et al. | End-to-end spatiotemporal CNN-LSTM network | ADNI dataset (*N* = 413 total: 140 CN, 80 EMCI, 93 LMCI, 34 MCI, 66 AD) | • Binary/Multi-class: ≥ 99% accuracy & ≥ 99.5% average AUC  • 3-Class (AD vs. MCI vs. CN): 98.99% accuracy | Relies on stringent noise scrubbing; highly susceptible to motion artifacts and spatiotemporal noise in real-world settings. | High Risk of Bias  (Single ADNI dataset without external clinical cohort validation; suspiciously near-perfect performance suggests implicit overfitting or sequence leakage) | TRL 3 (Low) |
|  | Moghaddami et al. | Graph Neural Network＋Recurrent Neural Network | *N*=303 subjects total. | • Next-Visit Decline: 82.9% prediction accuracy  • CN-to-MCI Conversion: 68.8% accuracy (early-stage task) | Relies heavily on complex non-Euclidean topological processing, limiting routine clinical workstation integration. | High Risk of Bias  (Validated on a small single retrospective cohort; vulnerable to attrition bias and lacks independent prospective external validation) | TRL 3 (Low) |
| sMRI (3D T1-w) + Clinical/Genetic Data | Ren et al. | Pre-trained Med3D + NMF + K-means + Multimodal LSTM with Attention | ADNI-2 dataset (*N* = 453 subjects with longitudinal follow-up) | Stratification & Trajectory Forecasting:  • 3 prognostic subgroups ("Low," "Mild," "Fast")  • Cognitive MAE: 0.196 (6m), 0.203 (12m), 0.261 (48m)  • Accuracy: 90.3% (6m), 84.5% (12m), 79.1% (48m) | Predictive accuracy decays significantly over longer horizons (drops to 79.1% at 48 months); lacks prospective external cohort validation in real-world settings. | High Risk of Bias  (Validated solely on a single retrospective ADNI-2 cohort; lacks prospective external validation) | TRL 3 (Low) |
| sMRI Segmentation + Clinical + Psychological Data | Jahan et al. | Random Forest (RF) Classifier + SHAP (Explainable AI / XAI) | OASIS-3 dataset  (5-class task: CN, AD, non-AD dementia, uncertain dementia, other dementia) | 5-Class Staging (OASIS-3):  • 10-fold CV Acc: 98.81% (RF vs. 8 baselines) | Uses pre-extracted FreeSurfer features over raw 3D images; lacks prospective external validation on OOD clinical cohorts to verify the 98.81% CV accuracy. | High Risk of Bias  (Single retrospective repository validation; potential subject-level longitudinal data leakage if split is session-based rather than patient-based; no external cohort test) | TRL 3 (Low) |
| sMRI + DTI | Marzban et al. | Shallow CNN fusing structural and diffusion metrics | ADNI dataset  • *N*=406 total scans  • Healthy Controls (HC): 185 scans  • MCI: 106 scans  • AD: 115 scans | AD vs. HC Classification (stacked MD＋GM volume):  • Accuracy: 93.5%  • AUC: 0.94  • Sensitivity: 92.5% / Specificity: 93.9% | Demands extensive preprocessing standardization to mitigate multi-scan variability across different scanners. | High Risk of Bias  (Validated solely on retrospective ADNI data without independent external clinical cohort testing) | TRL 3 (Low) |
|  | Li et al. | MADNet (Dual-branch ResNet-10) with Grad-CAM | Pooled multi-center dataset: ADNI + XWNI (Xuanwu Hospital)  (*N* = 185 total: 66 AD, 40 MCI, 79 CN) | AD vs. CN accuracy: 96.54% (AUC: 0.9597)  MCI early-warning accuracy: 83.33% (Specificity: 0.9347) | Demands extensive preprocessing standardization to mitigate multi-scan variability across different scanners. | High Risk of Bias  (Small sample size *N* = 185; combined ADNI+XWNI cohort split without strict prospective external cohort validation) | TRL 3 (Low) |
| PET | Choi et al. | Deep 3D Convolutional Neural Network | Retrospective cohort from the ADNI database (*N*=492 total baseline PET volumes) | MCI to AD conversion prediction accuracy: 84.2% | Heavily relies on standardized repositories; generalizability under high clinical scan heterogeneity is unvalidated. | High Risk of Bias  (Single retrospective ADNI cohort split without strict independent external prospective validation) | TRL 3 (Low) |
| Neurological Tests | Jasodanand et al. | Multimodal computational framework | 7 independent cohorts (*N*=12,185) | Aβ Classification AUROC: 0.79  Tau (τ) Classification AUROC: 0.84 | Framework requires extensive data harmonization; unvalidated in un-screened real-world community populations. | Low Risk of Bias  (Large multi-center sample *N* = 12,185 across 7 cohorts with independent external validation on ADNI, HABS, and NACC) | TRL 4 (Moderate) |
| Clinical + Blood + sMRI | Karlsson et al. | Predictive regression models (substitute for Tau-PET) | Multi-center cohorts (*N* > 2,500+ total):  • Primary: BioFINDER-2 (*n* = 1,195)  • External: BioFINDER-1 (*n* = 147), ADNI, UCSF-ADRC, OASIS3, A4 | Total Tau-PET Load Prediction: R^2^＝0.66–0.69  Hemispheric Asymmetry Capture: R^2^＝0.28–0.42 | Dependent on analytical sensitivity and global standardization of plasma assays; cross-sectional nature cannot track dynamic accumulation. | Low Risk of Bias  (Large multi-center sample *N* > 2,500 with rigorous independent validation across BioFINDER-1, ADNI, UCSF-ADRC, OASIS3, and A4 cohorts) | TRL 4 (Moderate) |
| Plasma Metabolomics | Stamate et al. | XGBoost Classifier (vs. standard DL and Random Forest) | High-dimensional cohort (*N*=357) | XGBoost optimal AUC: 0.88  DL & Random Forest AUC: 0.85  Sample size: *N*＝357 | Limited sample size (*N*=357) challenges generalization boundaries within extreme high-dimensional feature spaces. | High Risk of Bias  (Small cohort, relies on internal cross-validation/Monte Carlo sampling without independent external validation) | TRL 3 (Low) |
| Multimodal (Clinical + sMRI + APOE) | Yi et al. | XGBoost-SHAP Framework | Multi-center cohorts (*N* = 896 total):  • Primary/Internal: ADNI (*N* = 547: 189 NC, 302 MCI, 56 AD; 27 features)  • External: NACC (*N* = 349: 171 NC, 70 MCI, 108 AD; 9 features) | 3-class Acc / AUC: ADNI (87.57% / 0.91), NACC (80.52% / 0.88). SHAP identified CDRSB, ADAS13, and ventricle volume as risk factors, and MMSE/LDELTOTAL as protective. | External validation restricted to 9/27 features; lacks fluid biomarkers (plasma/CSF) and PET staging. | Low Risk of Bias  (Algorithmic sample weighting avoids resampling leakage; multi-center 3-class evaluation validated on independent external NACC cohort) | TRL 4 (Moderate) |
| Blood (RNA/SNPs), Clinical & PET Imaging | AlMansoori et al. | Multi-modal ML Pipeline (SVM + Mutual Information for combined data; RF & AdaBoost for single modalities; SHAP feature attribution) | ADNI Cohort  (*N* = 623 total; age-stratified analysis *n* = 620) | Early AD / MCI Detection:  • Combined Model (SVM+MI): Acc = 0.95, AUC = 0.94  • Single Modalities: Gene expression AUC = 0.65; SNPs AUC = 0.63 | No external validation (ADNI only); blood genetics alone show modest power (AUC 0.63–0.65); requires transcriptomic protocol standardization. | High Risk of Bias  (Single-database evaluation without independent external cohort validation) | TRL 3 (Low) |
| Brain Transcriptomics (Microarray) + Immune Infiltration Profiles (ssGSEA) | Lai et al. | ssGSEA + WGCNA + LASSO + XGBoost Framework (SHAP & LIME interpretability; benchmarked 6 ML models; unsupervised consensus clustering for subtyping) | 7 GEO Brain Datasets (*N* = 1,212 total): Discovery *n* = 436 (247 NC / 189 AD); External *n* = 776. | AD vs. NC Classification (XGBoost):  • Testing Set: AUC = 0.86, P-R AUC = 0.83 | Relies on post-mortem brain tissues rather than biofluids; retrospective microarray analysis with moderate AUC (0.86). | Moderate Risk of Bias  (Retrospective post-mortem microarray data, balanced by validation across 4 independent GEO sets and RT-PCR experiments) | TRL 3 (Low) |
| CSF Proteomics | Bader et al. | Mass spectrometry -based workflow & ML | 3 independent studies (*N*=197) | Quantified＞1,000 proteins (CV＜20%)  Identified alterations in tau, SOD1, PARK7  Sample size: 3 independent studies (*N*＝197) | Metabolic stress and accelerated glycolytic signatures lack unique pathological specificity to exclude non-AD dementias. | Low Risk of Bias  (Validated via a multi-center "rectangular strategy" across 3 independent cohorts with robust analytical reproducibility) | TRL 3 (Low) |
|  | Hou et al. | ML feature selection (12-protein panel) | Training cohort: 297_CSF proteomics baseline dataset (*N*=297) | Universal cross-center differentiation (AD vs. MCI vs. FTD)  Sample size: 10 independent global cohorts | Mandatory reliance on invasive lumbar punctures creates substantial logistical barriers for scalable early community screening. | Moderate Risk of Bias  (Secondary analysis of public datasets with partial sample overlap, mitigated by multi-cohort, cross-platform & ELISA validation) | TRL 3 (Low) |
